# Ball motion and bubble ripples in the interaction of cavitation bubble-elastic ball-curved wall^☆^

**DOI:** 10.1016/j.ultsonch.2025.107348

**Published:** 2025-04-11

**Authors:** Yanyang Liu, Jing Luo, Lixin Bai, Jiankun Hu

**Affiliations:** a Department of General Surgery & Laboratory of Gastric Cancer, State Key Laboratory of Biotherapy/Collaborative Innovation Center of Biotherapy and Cancer Center, West China Hospital, Sichuan University, Chengdu, China; b Gastric Cancer Center, West China Hospital, Sichuan University, Chengdu, China; c State Key Laboratory of Hydraulics and Mountain River Engineering, Sichuan University, Chengdu 610065, China

**Keywords:** Cavitation bubble, Elastic ball, Curved wall, Bubble ripples, Jet

## Abstract

Elastic ball motion and cavitation bubble ripples in cavitation bubble-elastic ball-curved wall interaction was investigated experimentally using single-electrode periodic discharge bubble generation technology and high-speed photography. It was found that the hard ball undergoes a process of “push-pull-push-pull” as the dimensionless bubble-ball distance increases, while the elastic ball undergoes a process of “push-pull” in the same scenario. This is mainly due to the combined effects of the expansion ejection effect, the reverse thrust of liquid jet and the secondary Bjerknes force of cavitation bubble and its rebound bubble, which are strengthened or weakened. The radial vibration of the elastic ball causes a continuous secondary Bjerknes force attraction effect between the ball and the wall, similar to that between an acoustic bubble and a wall. In the interaction of “cavitation bubble-elastic ball-curved wall,” there is a state of equilibrium stability where the centerline of the “bubble-ball” coincides with the centerline of the “bubble-wall.” Both the ball and the bubble will move towards this equilibrium position. This is a result of the three forces with different starting and ending points—the “bubble-wall” secondary Bjerknes force, the “ball-wall” secondary Bjerknes force, and the “bubble-ball” interaction force—reaching a condition of equilibrium. The evolution of the cavitation bubble is usually dominated by toroidal jets, sometimes forming multi-layered nested toroidal jets (annular cylindrical jet). The surface tension waves of the bubble, the elastic modulus waves and the curvature waves of the elastic ball work together to form cavitation bubble ripples. Under the primary intensification of the bubble’s rapid collapse and the secondary intensification of the wall effect, the bubble ripples are reinforced, leading to the formation of multi-layered nested toroidal jets.

## 1 Introduction

Cavitation bubbles can cause damage to surfaces due to the formation of high-speed jets, a phenomenon widely studied across various fields including hydraulic engineering [1], marine engineering [2], industrial cleaning [3], and so on. In the medical field, cavitation bubbles find applications in various areas such as ultrasound tumor therapy [4], [5], drug delivery [6], [7] and ultrasonic lithotripsy [8]. Presently, much research focus on the effects of rigid planar walls on cavitation bubbles, given the simplicity of this configuration and its ability to illustrate the general impact of walls on cavitation dynamics. This is because, in most scenarios, the curvature of the walls is large enough to be approximated as planar relative to the cavitation bubble. Brujan et al. [9] utilized high-speed photography and numerical simulation to investigate the cavitation dynamics and jetting behavior of bubbles between two rigid planar walls. Huang et al. [10] quantitatively analyzed the bubble dynamics between the free surface and a rigid planar wall. Nevertheless, cavitation dynamics under curved boundary conditions often provides a more accurate reflection of real-world scenarios. Recently, there has been growing interest in the impact of elastic surfaces and particles on cavitation, particularly due to the abundance of elastic and membranous structures in the human body like blood vessels, which is crucial for advancing biological and medical research.

Curved and tubular boundaries are more commonly encountered in medical practice and industrial processes compared to flat surfaces. For example, the microjets generated by bubble collapse in blood vessels can cause physical damage to blood cells or other circulating cells. Xue et al. [11]observed through numerical simulations that cavitation bubble collapse near a curved wall produces a high-speed jet capable of damaging the wall. Chen et al. [12] used high-speed photomicrography to observe ultrasound-induced bubbles within ex-vivo rat mesentery tissues. Their results demonstrated that blood vessels tend to cave in rather than bulge out, with liquid jets being directed away from the nearest vessel wall. Ma et al. [13] explored the relationship between boundary curvature and bubble dynamics using three hemispheres of different curvatures and stable spark-generated bubbles of the same size, identifying four typical bubble shrinkage patterns. Tomita, et al. [14] studied the growth and collapse of laser-induced bubbles under both convex and concave boundaries, combining both theoretical and experimental approaches. Li et al. [15] experimentally studied the bubble morphology, period, and microjets in tubes of different length, diameter, and the distance of the bubble-tube port varies. Their findings indicated that the cavitation bubble period would increase with the length of the tube but would first decrease and the stabilize as tube diameter increases. Aganin et al. [16] examined the dynamics of bubbles near a rigid wall with localized axisymmetric convex or concave unevenness using the boundary element method, noting significant variations in bubble shape as the convex radius changes. Dadvand et al. [17] carried out a numerical study using the boundary integral method to explore the three-dimensional dynamics of a transient bubble located in the corner formed by two rigid curved parabolic plates.

Spherical walls represent another form of curved boundary. Li et al. [18] combined high-speed photography and auxiliary function method simulation to study the nonlinear interaction between a bubble and a suspended sphere. In a subsequent paper, Li et al. [19] discussed the situation of a bubble and a movable sphere of comparable size near a rigid wall, showing that the bubble's dynamic behavior is strongly influenced by two dimensionless standoff parameters: one is the minimum distance between the sphere surface and the bubble center, and the other is the distance between the bubble center and the rigid wall. Shahalami et al. [20] investigated the dynamics of relatively small bubbles ranging from 1 mm to 3 mm in size near a 4.5 mm diameter glass sphere, utilizing an integrated thin film drainage apparatus (ITFDA) to measure forces and spherical deformation. Wang et al. [21] and Zheng et al. [22] experimentally and theoretically analyzed the bubble dynamics near one sphere or two same-sized spheres, predicting the bubble centroid movement based on whether the initial position is along the horizontal symmetry axis of the two particles or not. In addition, many researchers have also studied the interaction between cavitation bubbles and solid balls [23], [24], [25], [26], [27].

Rigid curved boundaries are prevalent in industrial practice, whereas elastic boundaries or particles are more common in medical and biological fields [28], [29]. Gibson [30] first observed the migration and outward ejection of microjets as bubbles expand and collapse near elastic boundaries. This prevents cavitation erosion because the soft surface repels the bubbles, thus mitigating or even hindering jet impact. Brujan et al. [31] focused on the interaction between a laser-induced cavitation bubble and an elastic boundary, and discussed the general principles of forming annular and axial jets. They discovered that the jetting behavior rresults from the interaction between the counteracting forces induced by the rebound of the elastic boundary and the Bjerknes attraction towards the boundary. In a subsequent study, Brujan et al. [32] varied the elastic modulus of the elastic boundary to mimic various biological tissues, such as renal parenchyma, abdominal aorta, femoral artery, articular cartilage, muscle, and cornea. Their experiments concluded that liquid jet penetration into the elastic boundary, jet-like ejection of boundary material, and tensile-stress-induced deformations of the boundary are the major mechanisms responsible for cavitation-enhanced ablation of elastic materials and cavitation erosion. Xu et al. [33] also proposed that different elastic moduli exert various repulsive effects on the spark-induced bubble. Also, the bubble's potential to penetrate the boundary and its interaction with the elastic boundary are strongly related to the elastic modulus. Our previous study [34] observed the interaction between cavitation bubbles and elastic vessel models with different wall thickness. We found that with the increase of the thickness of the blood vessel wall, the shape and collapse time of the cavitation bubble first increased and then tended to be stable, and the stretching amplitude of the blood vessel wall caused by bubble expansion was greater than that caused by contraction. Ma et al. [35] compared the elastic and rigid planar boundaries and found that the bubble tends to form a mushroom shape near the elastic boundary due to boundary deformation and rebound. However, the bubble always migrates towards the boundary with a high-speed jet when it is created near a rigid boundary. As to the curved elastic boundaries, Liu et al. [36] set up a numerical model and investigate the potential for a cavitation bubble to cause mechanical damage on the curved elastic boundary, but lacks experimental verifications.

Cavitation bubbles can also affect particle motion [37], [38], [39], [40], [41], [42], [43]. Poulain et al. [44] concluded that a spherical particle is pushed away when the bubble grows and the particle then approaches the bubble during and after collapse. Lv et al. [45] discussed the influence of the bubble-particle distance and the particle/bubble radius ratio on the laser-induced cavitation bubble's effect on dense SiO2 particles. In general, the distance between the particle and the bubble significantly affects the particle’s motion. When the distance between the two is close, the particle tends to move away from the bubble, and when the distance between the two is far, the particle tends to move closer to the bbubble. Ren et al. [46] reported that millimeter-sized stationary spherical particles can be suddenly accelerated by the generation of nearby laser-induced cavitation bubble of similar size. Ma et al. [47] experimentally investigated that a series of particles in the Acoustic Lichtenberg Figure (ALF) cavitation structure tend to transport along the bubble chain and move towards the focus repeatedly and the trajectory can be predicted.

From the review above, it is clear that the interactions between curved walls, particles, elastic boundaries, and cavitation bubbles have been explored by some scholars. However, research on the movement of particles is not detailed enough. Given the potential medical applications of this scenario, we conducted a detailed experimental study on the motion of elastic balls in cavitation bubble-elastic ball-curved wall interactions, and from the authors’ knowledge, for the first time proposed the theory of bubble ripples to explain the formation of multi-layered nested toroidal jets.

## 2 Experiment

**Fig. 1:**
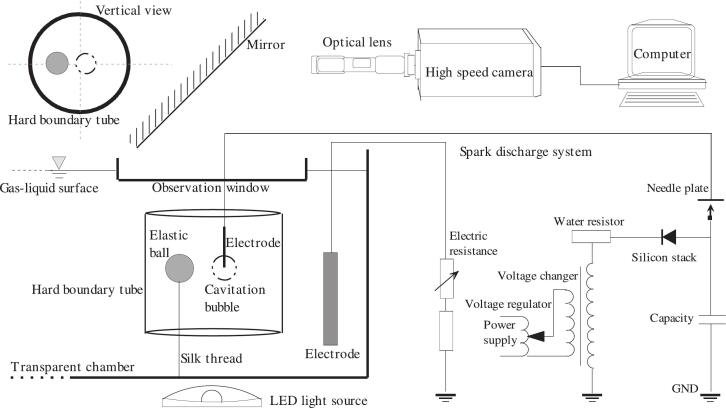
Experimental setup to visualize the interaction between a cavitation bubble and an elastic ball in hard boundary tubes.

Fig. 1 illustrates the experimental setup when the interaction between a cavitation bubble and an elastic ball in hard boundary tubes is recorded. The experimental setup consisted of a cavitation bubble generation system, the high-speed imaging and illumination system, and a device to adjust the relative position of the elastic ball, hard boundary tube and electrode, etc. Cavitation bubbles are generated using high-voltage spark discharge. The spark discharge bubble generation system adopts a combination of air discharge and water discharge, which can achieve periodic repeated discharge (27.5 kV, 15 Hz) and produce cavitation bubbles with good repeatability. A single electrode discharge bubble generation technique was adopted in the experiment, which can generate cavitation bubbles at the end of a 1.3 mm wire, reducing the interference with bubble deformation. The hard boundary tubes (diameter = 104 mm) are vertically fixed in a transparent water tank (500 mm × 260 mm × 300 mm). The small balls (an elastic rubber hollow ball and a hard boundary steel hollow ball, diameter = 25 mm, mass = 5.6 g) are fixed by the tension of a soft silk thread and their own buoyancy, and can only move freely in the horizontal direction, ensuring that the balls still remain in the same section of the hard boundary tube after movement. The deformation and motion of cavitation bubble and ball is recorded with a high-speed camera (Photron Fastcam SA-Z, Photron Ltd., Japan) from the top view. The pictures are taken in a framing rate of 20,000 fps (1024 × 1024 pixels and 20 µm × 20 µm pixel size). An organic glass observation window was used to eliminate the influence of gas–liquid interface fluctuations on observation.

## 3 Results

**Fig. 2:**
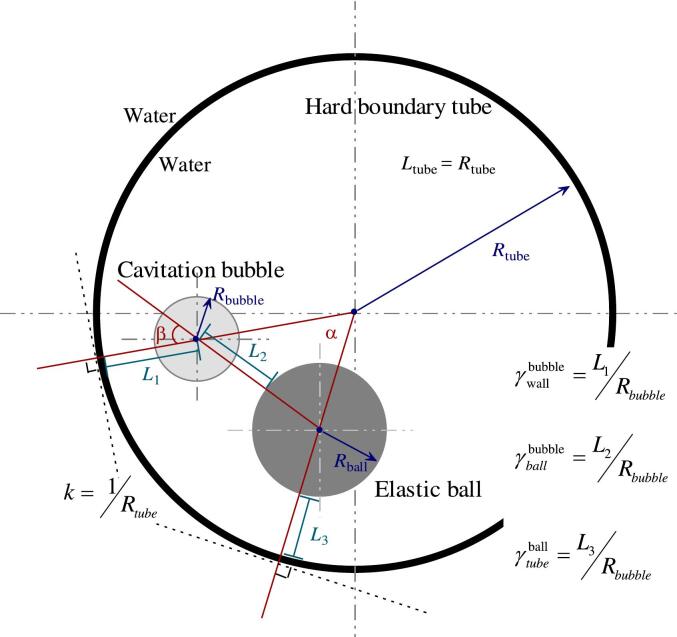
Position relationship between cavitation bubble, elastic ball, and hard boundary tube.

The three objects involved in this study—the elastic ball (spherical symmetry), the cavitation bubble (planar symmetry), and the tube (axial symmetry)—are all symmetric structures, and they are fixed in the same plane (the cross-sections of all three are in the same plane). However, it is still necessary to determine the positional relationship among them using three lengths and two angles. The dimensionless bubble-wall distance γtubebubble=L1Rbubble, dimensionless bubble-ball distance γballbubble=L2Rbubble, dimensionless ball-wall distance γtubeball=L3Rbubble, bubble-ball angle α, and ball-wall angle β are defined in the paper (as shown in Fig. 2). Here, $R_{\mathrm{bubble}}$ is defined as the radius when the cavitation bubble expands to its maximum volume; if the bubble does not form a perfect sphere at its maximum volume, $R_{\mathrm{bubble}}$ is the distance from the electrode to the farthest point on the arc (usually there is still an arc segment unaffected by the ball and the tube). $L_{1}$ is the shortest distance from the electrode to the tube wall, which is the distance from the bubble center to the intersection point of the line connecting the bubble center and the tube center with the tube wall; when$L_{1}$ = 0, it indicates that the electrode is in contact with the tube wall and the bubble is generated by discharge right on the wall. $L_{2}$ is the shortest distance from the electrode to the ball's surface, which is the distance from the bubble center to the intersection point of the line connecting the bubble center and the ball center with the ball's surface; when$L_{2}$ = 0, it indicates that the electrode is in contact with the ball's surface and the bubble is generated by discharge right on the ball's surface. $L_{3}$ is the shortest distance from the surface of ball to the tube wall, which is the distance between the intersection points of the line connecting the ball center and the tube center with the tube wall and with the ball wall; when$L_{3}$ = 0, it indicates that the ball is in contact with the tube wall. α is the angle between the line connecting the bubble center and the tube center and the line connecting the ball center and the tube center. β is the angle between the line connecting the bubble center and the tube center and the line connecting the bubble center and the tube center.

### 3.1 The motion of the ball in bubble-ball interaction

**Fig. 3:**
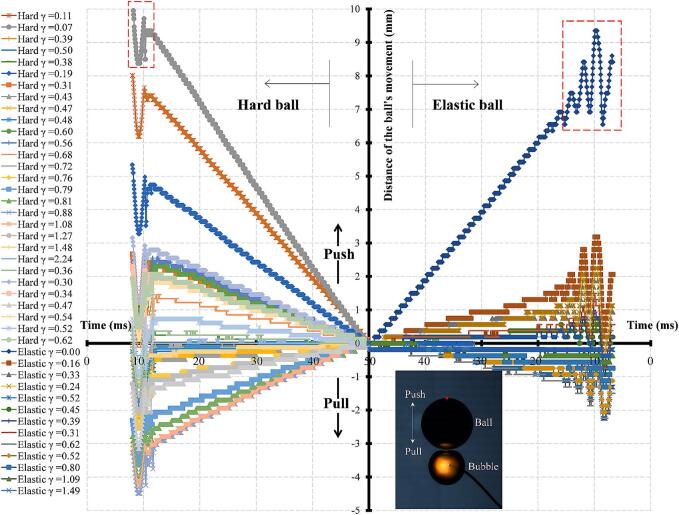
The variation of the movement distance of the farthest point on the ball wall from the bubble with time at different dimensionless bubble-ball distances.

**Fig. 4:**
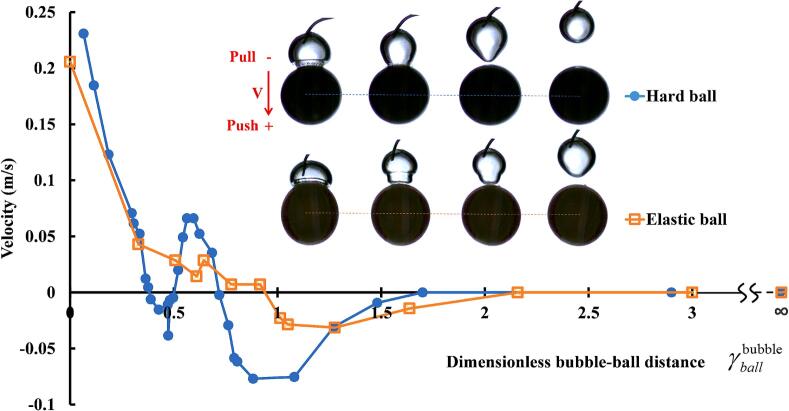
The variation of the ball's velocity with the dimensionless bubble-ball distance.

Before investigating the interaction of bubble-ball in a confined space like a tube, it is necessary to study the interaction of a cavitation bubble and an elastic ball (both of which are capable of deformation) in an unrestricted space. We prepared five different sizes of elastic balls for the experiment, but considering the objectives of this paper, only the most representative elastic ball with a diameter of 25 mm was selected for analysis. A hollow thin-walled elastic ball (made of rubber, with a wall thickness of 2 mm, an outer diameter of 25 mm, and a weight of 5.6 g) with a membrane structure similar to that of ultrasound contrast agents and red blood cells is used in this study. In addition to the inherent elasticity of the rubber material, the elasticity due to the deformation of the curved thin-walled structure and the elasticity produced by the gas enclosed within the rubber sphere all influence the deformation of the rubber sphere. To differentiate the effects of curvature and elastic deformation, a comparative study was conducted on a thin-walled rigid hollow ball of the same size and weight (made of stainless steel, with a wall thickness of 1 mm, an outer diameter of 25 mm, and a weight of 5.6 g). Fig. 3 shows the variation of the ball's movement over time at different dimensionless bubble-ball distances. The left quadrant represents the hard ball, the right quadrant represents the elastic ball, the upper quadrant represents being pushed away, and the lower quadrant represents being pulled closer. Since the curvature, buoyancy, and drag of the two types of balls are the same for the deformation of the cavitation bubble and the motion of the ball, the only difference between them is elasticity, thus a comparative study can be conducted. Although some of the curves in Fig. 3 are not clear, each curve has corresponding data points in Fig. 4.. For comparison, the direction of time on the left and right sides of Fig. 3 is opposite (symmetrical). It can be seen from the figure that the maximum distance the ball is pushed away is 3–4 times the maximum distance it is pulled closer. The hard ball's movement distance is more sensitive to the impact of the cavitation bubble, while the elastic sphere, except in the case where the electrode is contact with the ball's wall ($\gamma_{\text{ba}ll}^{\mathrm{bubble}}\approx 0$), its movement distance is not sensitive to the impact of the cavitation bubble, meaning the elastic ball tends to stay where it was. Due to the growth and collapse of the cavitation bubble, the ball is also pushed away and pulled closer, resulting positional shaking, as shown by the red dashed box in Fig. 3. The elastic ball, due to its elastic deformation (surface fluctuation), has a large amplitude and longer duration of vibration. This phenomenon can be explained from an energy perspective; for the hard sphere, all the energy released by the cavitation bubble is converted into the ball's macroscopic kinetic energy, so its response to impact is greater and more sensitive; for the elastic sphere, the energy released by the cavitation bubble is not only converted into the ball's macroscopic kinetic energy but also into the ball's own radial vibration and surface fluctuation energy, so it has a small and insensitive response to the impact of the cavitation bubble.

Because the movement distance of the ball changes almost linearly with time within the first 40 ms after the cavitation bubble impact, and only after that, the slope will significantly decrease, this indicates that the ball is moving at a constant velocity during the first 40 ms. The velocity of the ball under various conditions during this 40 ms period can be determined by the slope of the line in Fig. 3. According to the impulse principle, $I=F\cdot \text{t}=\text{m}\cdot v_{f}-m\cdot v_{i}$, where, *m* represents the mass of the ball, $v_{f}$ represents the final velocity of the ball, $v_{i}$ represents the initial velocity of the ball. Since the initial velocity is 0, and the duration of the bubble's first growth and collapse, which is the time during which the bubble exerts force on the ball, is stable between 2.2–2.3 ms, the magnitude of the force exerted by the bubble on the ball can be evaluated by the velocity.

Fig. 4. shows the variation of the ball's velocity with the dimensionless bubble-ball distance. The direction away from the ball is considered positive, while the direction towards the ball is negative. It can be seen that the hard ball experiences three changes in the direction of motion as the dimensionless bubble-ball distance increases (push–pull-push–pull), whereas the elastic ball only undergoes one changes in direction (push–pull). This phenomenon was first discovered in this study.

When the cavitation bubble is generated on the ball's wall ($\gamma_{\text{ba}ll}^{\mathrm{bubble}}\approx 0$), both the hard and elastic balls are pushed away, and their ejection velocities are at their maximum (the hard ball's velocity is slightly greater than that of the elastic ball). As the dimensionless bubble-ball distance increases ($\gamma_{\text{ba}ll}^{\mathrm{bubble}}\approx 0∼0.3$), the velocity of both balls decreases rapidly. As the dimensionless bubble-ball distance continues to increase ($\gamma_{\text{ba}ll}^{\mathrm{bubble}}\approx 0.3∼1$), there is a significant difference in the motion of the hard and elastic balls under the influence of the bubble. For the hard ball: In the interval ($\gamma_{\text{ba}ll}^{\mathrm{bubble}}\approx 0.4∼0.5$), the hard ball changes its direction of motion, moving towards the bubble, with the velocity first increasing and then decreasing, reaching an extremum at the midpoint of the interval, with a relatively small absolute value of velocity. In the interval ($\gamma_{\text{ba}ll}^{\mathrm{bubble}}\approx 0.5∼0.7$), the ball changes direction again, moving away from the bubble, with the velocity first increasing and then decreasing, reaching an extremum at the midpoint of the interval, with a relatively large absolute value of velocity. In the interval ($\gamma_{\text{ba}ll}^{\mathrm{bubble}}\approx 0.7∼\infty$), the ball changes direction once more, moving towards the bubble, with the velocity first increasing and then decreasing, reaching an extremum near $\gamma_{\text{ba}ll}^{\mathrm{bubble}}\approx 1$. For the elastic ball: In the interval ($\gamma_{\text{ba}ll}^{\mathrm{bubble}}\approx 0.3∼0.9$), the ball's velocity does not change, and the absolute value of the velocity is relatively small. In the interval ($\gamma_{\text{ba}ll}^{\mathrm{bubble}}\approx 0.9∼\infty$), the ball changes direction for the first time, moving towards the bubble, with the velocity first increasing and then decreasing, reaching an extremum near $\gamma_{\text{ba}ll}^{\mathrm{bubble}}\approx 1.2$. It can be seen from Fig. 4. that the direction and velocity of the ball's motion, or the direction and magnitude of the force exerted by the bubble on the ball, are closely related to the deformation of the bubble.

When the cavitation bubble is very close to the ball, it undergoes an explosive spherical expansion, generating an ejection effect on the ball. The closer the bubble is to the ball, the more pronounced the ejection effect. When the electrode end is on the ball's wall ($\gamma_{\text{ba}ll}^{\mathrm{bubble}}\approx 0$), the outward thrust on the ball reaches its maximum. After the ejection, the bubble begins to collapse nonspherically, producing a toroidal jet and forming a mushroom-shaped bubble, which has been mentioned in previous literature. The bubble's collapse does indeed exert a pulling force on the ball, but because the collapse is not spherically symmetric, the bubble collapses faster along the circumference of the line connecting the ball's center, so the pulling force from the nonspherical collapse is smaller compared to the thrust generated during the spherical expansion of the bubble. When the mushroom-shaped bubble collapses to its final state, the toroidal jets collide at a certain angle in a “V” shape, producing an axial jet. Depending on the different dimensionless bubble-ball distances, i.e., the different ratios of the toroidal jet velocity and the bubble collapse velocity, it may only produce a jet moving away from the ball, or only a jet moving towards the ball, or both jets moving away from and towards the ball, but the strengths of the two jets differ under different conditions. When $\gamma_{\text{ba}ll}^{\mathrm{bubble}}\approx 0$, the bubble collapse only produces a jet moving away from the ball, regardless of whether it is a hard ball or an elastic ball. According to the conservation of momentum, in the axial direction, as the jet moves away from the ball, the ball must move in the opposite direction of the jet, and the stronger the jet, the greater the ball's movement speed in the opposite direction. Combining the analysis of the three stages (expansion phase, collapse phase, and post-collapse jet phase), when $\gamma_{\text{ba}ll}^{\mathrm{bubble}}\approx 0$, the ball is pushed away by the bubble, and the velocity is at its maximum.

For the hard ball, as the dimensionless bubble-ball distance increases, the outward ejection effect produced by the bubble expansion weakens, while the hard ball, acting as a hard boundary, exerts a stronger secondary Bjerknes attraction on the bubble. At the same time, the toroidal jet produced by the asymmetric collapse of the bubble, which moves away from the ball, weakens, while the microjet towards the wall surface of the bubble strengthens. When both jets act together, at $\gamma_{\text{ba}ll}^{\mathrm{bubble}}\approx 0.47$, it manifests as a jet directed towards the ball. According to the conservation of momentum, when the jet is directed towards the ball, the ball must move in the opposite direction of the jet, that is, towards the direction of the bubble. Considering the three effects (expansion ejection effect, secondary Bjerknes effect, and jet effect) together, when $\gamma_{\text{ba}ll}^{\mathrm{bubble}}\approx 0.47$, the ball is attracted by the bubble and moves towards the bubble.

For the hard ball, as the dimensionless bubble-ball distance continues to increase, the influence of the bubble's initial growth and collapse on the ball weakens, while the influence of its rebound bubble's expansion and collapse on the ball strengthens. When $\gamma_{\text{ba}ll}^{\mathrm{bubble}}\approx 0.56$, the position of the rebounding bubble and its effect on the ball are similar to the situation when $\gamma_{\text{ba}ll}^{\mathrm{bubble}}\approx 0$. The ejection effect and the reverse thrust effect of liquid jet of the rebounding bubble are the strongest, and the ball's velocity away from the bubble reaches a local extreme value.

For the elastic ball, when $\gamma_{\text{ba}ll}^{\mathrm{bubble}}\approx 0.3∼0.7$, there is no change in the direction of the ball's motion or a dramatic change in the ball's velocity because the elastic boundary weakens the secondary Bjerknes effect and the microjet effect. Specifically, the expansion and contraction of the elastic ball and the bubble are not exactly opposite, so there is a secondary Bjerknes attraction between the bubble and the elastic ball, similar to that between vibrating bubbles. At the same time, the elastic boundary causes the bubble to collapse in the opposite direction, and these two effects cancel each other out, weakening the macroscopic motion of the elastic ball.

For both the hard ball and the elastic ball, when $\gamma_{\text{ba}ll}^{\mathrm{bubble}}>1$, the secondary Bjerknes force becomes the dominant force between the bubble and the ball, causing the ball to move towards the bubble. As the distance increases, the interaction between the two becomes weaker, and the ball's movement velocity decreases, eventually reaching zero.

### 3.2 The motion of the ball and the deformation of the bubble in bubble- ball-wall interaction

**Fig. 5:**
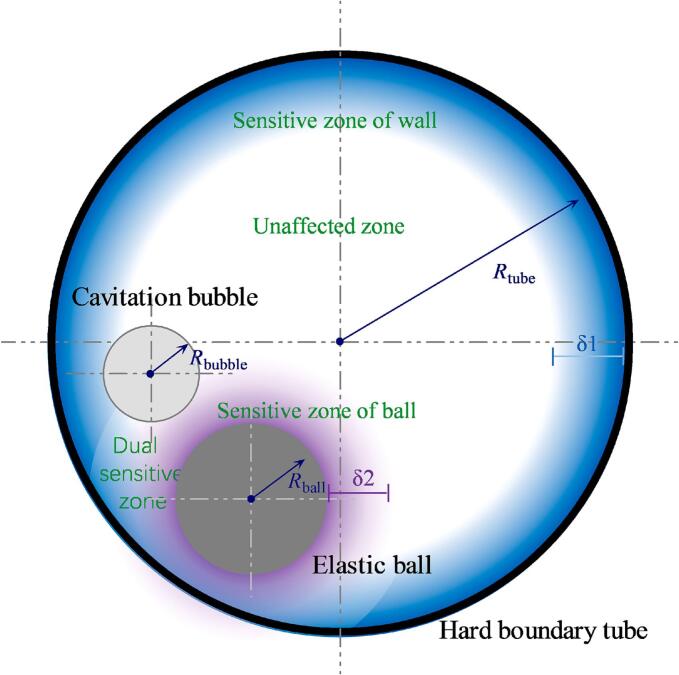
Schematic diagram of the interaction of cavitation bubble-elastic ball-hard boundary tube.

The interaction between cavitation bubble and elastic ball is related to the dimensionless bubble-ball distance. According to different evaluation criteria (such as bubble deformation, changes in the position of the rebounding bubble, movement of the ball, or surface oscillations of the ball), we can always find a bubble-ball distance *δ*1 beyond which (varying according to different standards, this threshold distance may differ), the ball will no longer be affected by the cavitation bubble. Similarly, based on different criteria (the sphericity of the bubble when it expands to its maximum volume, the deformation during the bubble collapse, the degree of deviation from the discharge position when the bubble collapses to its minimum volume, etc.), there exists such a bubble-wall distance threshold *δ*2 for the wall's influence on the bubble. Accordingly, we can divide the interaction of the bubble-ball-tube into four regions: the unaffected zone, the wall-sensitive zone, the elastic ball-sensitive zone, and the dual-sensitive zone of the wall and elastic ball (as shown in Fig. 5). According to this classification, three typical distributions were investigated:

Distribution-1: γtubebubble<δ1Rbubble, γballbubble>δ2Rbubble, γtubeball<L3′Rbubble;

Distribution-2: α=0, β=0, γtubebubble<δ1Rbubble, γballbubble<δ2Rbubble;

Distribution-3: α≠0, β≠0, γtubebubble<δ1Rbubble, γballbubble<δ2Rbubble.

#### 3.2.1 Distribution-1

**Fig. 6:**
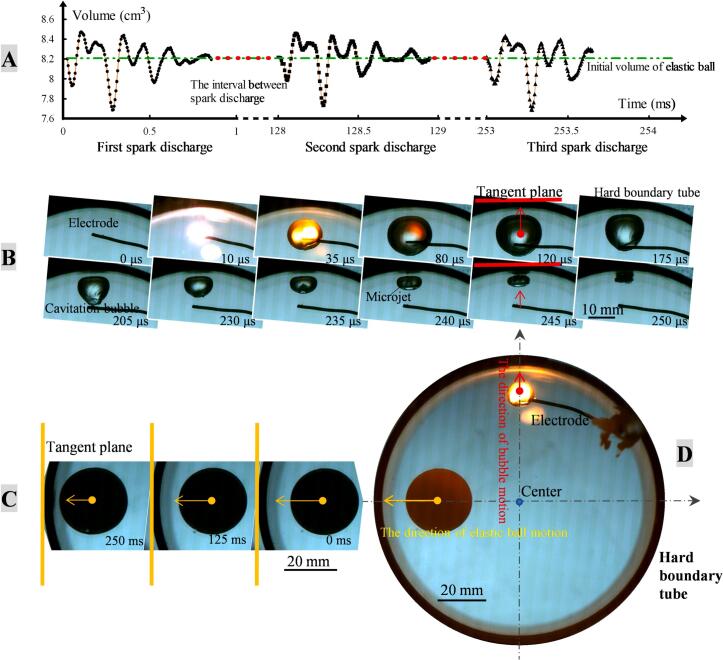
The interaction between elastic ball and curved wall ($\gamma_{\mathrm{tube}}^{\text{bubble}}=0.97$, $\gamma_{\mathrm{ball}}^{\text{bubble}}=3.93$, $\gamma_{\mathrm{tube}}^{\text{ball}}=0.91$, α=90°, β=38°). (A) The volume of the elastic ball in subfigure (D) changes over time; (B) The growth and collapse of cavitation bubble in subfigure (D); (C) The motion of the elastic ball in subfigure (D); (D) The distribution of the elastic ball and the cavitation bubble in the tube.

The interaction between cavitation bubbles and curved walls, as well as between cavitation bubbles and elastic balls, has been a focus of previous research, but the interaction between elastic balls and curved walls tends to be overlooked. In fact, under the action of cavitation bubble, the elastic ball will vibrate radially, thereby exhibiting macroscopic movement behaviors similar to acoustic bubbles. To investigate the interaction between elastic balls and curved walls, we set the bubble-ball angle *α* to 90 degrees (as shown in Fig. 6(D)). The cavitation bubble and the elastic ball approach the tube surface (γtubebubble<δ1Rbubble, γtubeball<L3′Rbubble). The bubble and the ball are far apart (γballbubble>δ2Rbubble), and the deformation of the bubble is not influenced by the ball, but is strongly affected by the curved wall. The bubble moves towards the tangent plane of the curved wall along the radial direction (as shown in Fig. 6(B)), which is consistent with the result drawn in the previous section of this paper. The pressure waves generated by the growth and collapse of the cavitation bubble can compress and dilate the elastic ball, causing it to vibrate radially (as shown in Fig. 6(A)). To amplify the ball's movement effect, periodic bubble generation technology was used to produce bubbles of the same size at the electrode end at intervals of 125 ms. It can be seen from the figure that the radial vibration of the ball shows good repeatability under the impact of three collapses of the bubble.

**Fig. 7:**
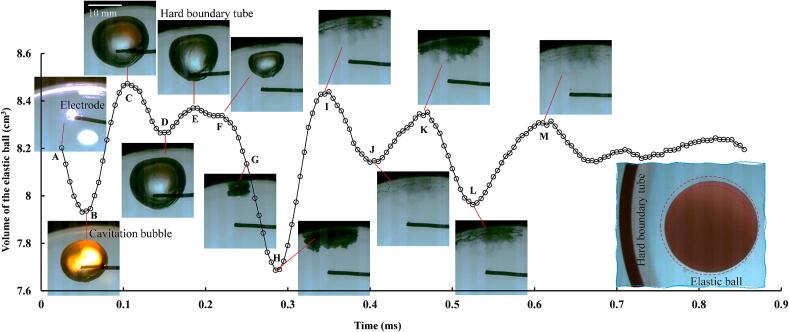
The correlation between cavitation bubble vibration and elastic ball vibration.

Fig. 7 shows the phase relationship between cavitation bubble deformation and elastic ball vibration. When the spark discharge generates a cavitation bubble (point A), a primary shock wave is produced simultaneously, compressing the elastic ball. When the bubble expands to about half of its maximum volume, the ball is compressed to its minimum value (point B), and then begins to rebound under its own elasticity. At the same time, the negative pressure following the primary shock wave also aids in the ball's expansion. When the bubble reaches its maximum value, the ball's volume also reaches its maximum. Subsequently, the ball begins to contract under its own elasticity (point D), while the bubble remains near its maximum volume, indicating that the pressure wave did not participate in the compression process of the elastic ball. Afterward, as the bubble begins to collapse, the negative pressure causes the ball to slightly expand (points E and F) before continuing to contract. After the bubble collapses to its minimum volume (point G), the pressure wave continues to compress the ball, reducing its volume. It is not until the rebounding bubble expands to its maximum volume that the ball reaches its minimum volume (point H), indicating that inertia causes the ball's vibration to lag behind the pressure wave. The ball then undergoes several expansion and contraction processes (points I, J, K, L, M), as the pressure stimulation from the bubble is very weak, and these vibrations of the ball should be due to the ball's elastic force. It can be seen from the figure that the vibration of the elastic ball has a significant periodicity, and this periodic vibration behavior similar to acoustic bubbles will cause the elastic ball to exhibit translational movement behavior similar to that of an acoustic bubble. The elastic ball moves radially towards the tangent plane direction of the hard boundary tube wall under the repeated impact of bubbles, and eventually hits the wall and rebounds (as shown in Fig. 6 (C)), indicating that its driving force is the secondary Bjerknes force.

**Fig. 8:**
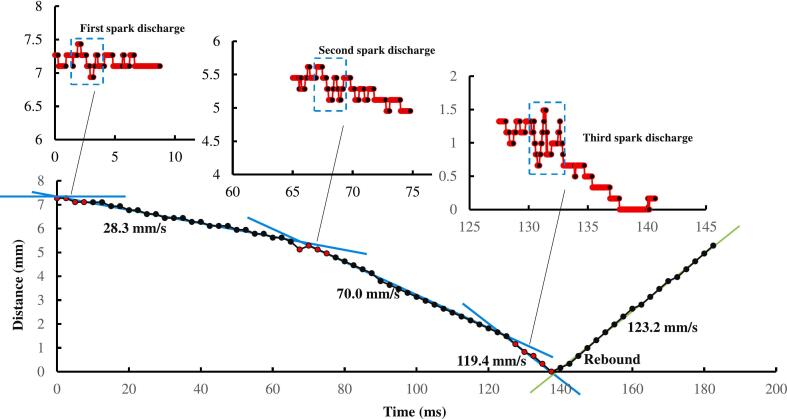
The motion of the elastic ball under three impacts of cavitation bubbles.

We measured the motion parameters of the elastic ball (as shown in Fig. 8) and found that the velocity of the ball increased after each cavitation bubble impact. After the first impact of cavitation bubble, the ball's velocity increased from 0 mm/s to 28.3 mm/s, after the second impact it increased from 28.3 mm/s to 70.0 mm/s, and after the third impact it increased from 70.0 mm/s to 119.4 mm/s. According to the principle of momentum, impulse is equal to the change in momentum, and the impulses for the three impacts can be calculated as 1.59 × 10^−4^ Ns, 2.33 × 10^−4^ Ns, 2.77 × 10^−4^ Ns, respectively. The closer to the wall, the greater the impulse, and the duration of action is about 10 ms, so the secondary Bjerknes forces produced by the elastic ball after three bubble impacts can be calculated as approximately 1.59 × 10^−2^N, 2.33 × 10^−2^N, 2.77 × 10^−2^N, respectively. This specific case confirms the existence of the secondary Bjerknes force acting on the elastic ball. In fact, the trend of elastic ball moving towards the wall is a very common phenomenon in experiments. When investigating the interaction between cavitation bubbles, elastic balls and walls, if the focus is on translational motion, the secondary Bjerknes force on the elastic ball is a factor that should not be ignored.

#### 3.2.2 Distribution-2

**Fig. 9:**
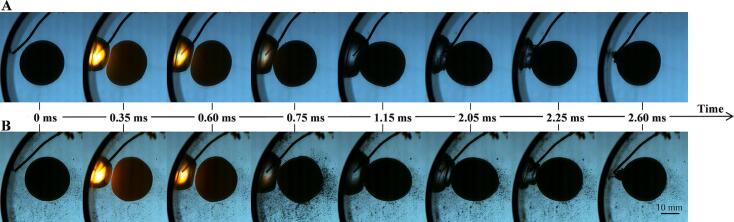
The repeatability of bubble deformation under complex boundary conditions. (A) $\gamma_{\mathrm{ball}}^{\text{bubble}}=0.42$, $\gamma_{\mathrm{tube}}^{\text{bubble}}=0.00$; (B) $\gamma_{\mathrm{ball}}^{\text{bubble}}=0.48$, $\gamma_{\mathrm{tube}}^{\text{bubble}}=0.00$.

The distribution where the cavitation bubble and the elastic ball are far apart was discussed in the previous section. The distribution where the cavitation bubble is close to both the elastic ball and the hard boundary tube (the bubble is in the ball-sensitive zone as well as in the wall-sensitive zone) will be discussed in the following two sections. Bubbles are affected by both the curved wall and the elastic ball, resulting in significant and complex dynamic deformation. The reproducibility of the experiment is a prerequisite for the study. The single-electrode discharge technology was used to reduce the interference of the electrodes on the bubble deformation. In addition, the periodic discharge technology was used to obtain a large amount of data in a short time, minimizing the impact of external environmental conditions. It can be seen from Fig. 9 that for the same relative positions of the bubble, ball and wall, the entire physical process (deformation and motion of the bubble and ball) can be reproduced perfectly in both time and space. In addition, comparing Fig. 9(A) and Fig. 9(B), it can be found that the residual microbubbles after the previous spark-induced cavitation bubble collapse do not affect the deformation of the cavitation bubble and the elastic ball.

**Fig. 10:**
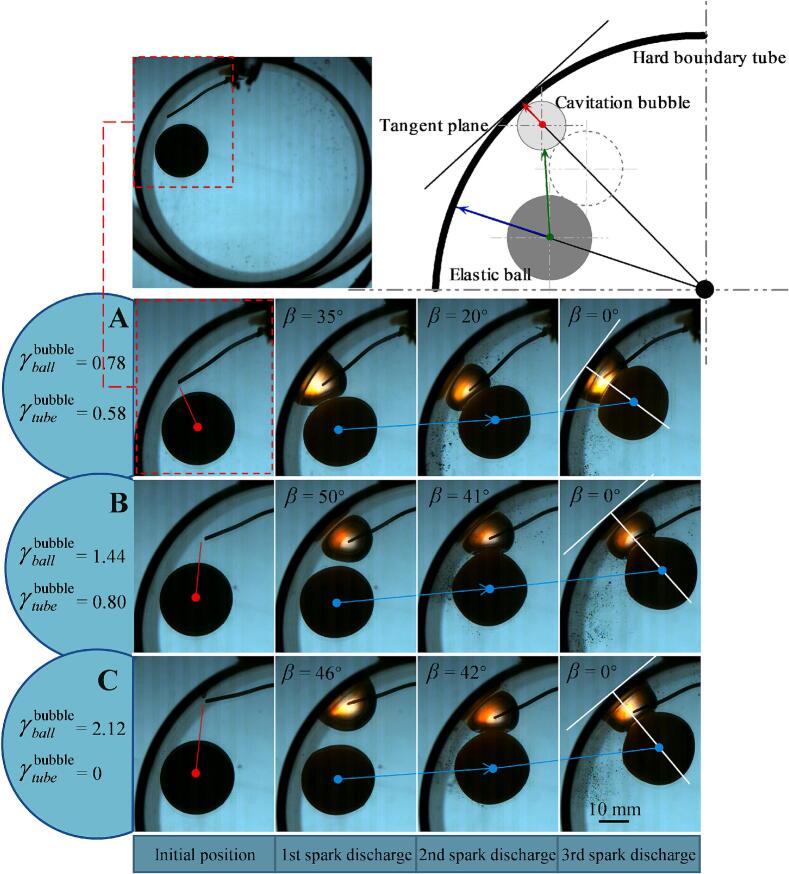
The equilibrium position of the elastic ball in the interaction of bubble-ball-tube.

**Fig. 11:**
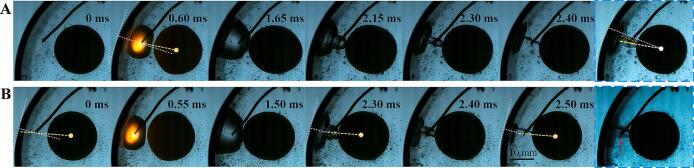
The equilibrium position of the cavitation bubble in the interaction of bubble-ball-tube. (A) $\gamma_{\mathrm{ball}}^{\text{bubble}}=0.54$, $\gamma_{\mathrm{tube}}^{\text{bubble}}=0.56$, *β* = 8°; (B), $\gamma_{\mathrm{tube}}^{\text{bubble}}=0.48$, *β* = 10°.

It is found that in the experiment of repeatedly generating bubbles through electric spark discharge, there is a special distribution that frequently occurs with a probability exceeding the general rate, where the centers of the bubble, the elastic ball, and the tube are collinear, with the bubble located between the ball and the tube wall (α=0, β=0, γtubebubble<δ1Rbubble, γballbubble<δ2Rbubble). At different dimensionless bubble-ball distances, the ball moved to the line connecting the centers of the bubble and the tube, forming a layout where the three centers are collinear (as shown in Fig. 10). When the positions of the tube and the bubble (electrode) are fixed, each growth and collapse of the cavitation bubble will cause the ball to move a certain distance, eventually reaching a position where the three centers are collinear. This indicates that this position is an equilibrium position. In the interaction between the cavitation bubble and the hard boundary tube, the bubble will collapse towards the tangent plane of the nearest point on the tube wall, that is, along the centerline of the bubble and the tube. There is a secondary Bjerknes force between the bubble and the tube wall. In the interaction between the cavitation bubble and the elastic ball, the ball moves towards the bubble, that is, along the line connecting the centers of the bubble and the ball, and there is an interaction force between the ball and the bubble. In the interaction between the elastic ball and the tube wall, the ball moves towards the tangent plane of the nearest point on the tube wall, that is, along the line connecting the center of the ball and the tube, and there is a secondary Bjerknes attraction between the ball and the tube wall. The three forces act together, and to achieve balance and stability, the directions of the three forces need to coincide. In this experiment, the ball is movable, the point on the cylinder wall closest to the ball changes with the position of the ball, and the position of the bubble is fixed, so the final equilibrium position of the ball is above the bubble on the line connecting the bubble and the tube wall. At this position, the ball can satisfy moving towards the wall is the same as moving towards the bubble, and vice versa. If the above reasoning is correct, then it can be inferred that if the position of the ball is fixed, the bubble will move towards the line connecting the center of the ball and the tube, and its final equilibrium position should be where the shortest distance between the ball and the tube wall. The experiment confirms that the bubble indeed tends to move towards this position, as shown in Fig. 11. When the ball-wall angle *β* is very small, the bubble tends to move towards the line connecting the center of the ball and the tube within a cycle of expansion and contraction. This indicates that this point is the equilibrium position of the cavitation bubble.

**Fig. 12:**
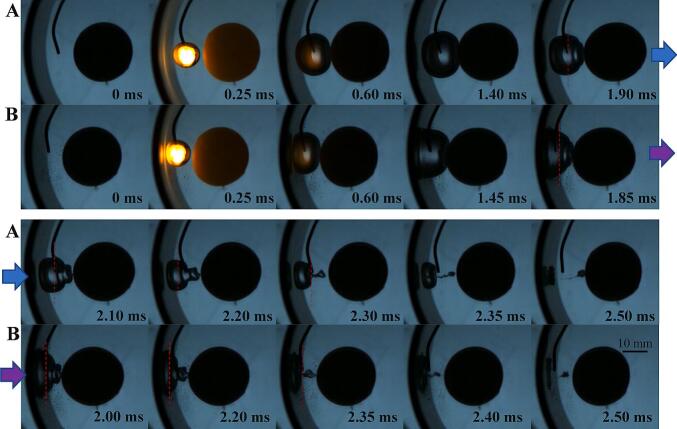
The proximity principle of bubble deformation. (A) $\gamma_{\mathrm{ball}}^{\text{bubble}}=0.60$, $\gamma_{\mathrm{tube}}^{\text{bubble}}=0.96$; (B) $\gamma_{\mathrm{ball}}^{\text{bubble}}=0.58$, $\gamma_{\mathrm{tube}}^{\text{bubble}}=0.48$.

In the equilibrium position of the three-center (bubble-ball-wall) collinear, the elastic ball and the hard tube wall are located on opposite sides of the cavitation bubble, with the region of the bubble wall most affected by the ball and the region of the bubble wall most affected by the wall being isolated by the bubble itself. In other words, the two sides of the bubble are respectively in the bubble-sensitive zone and the wall-sensitive zone. Fig. 12 compares two experiments with similar dimensionless bubble-ball distances and different dimensionless bubble-wall distances. It can be observed that if the bubble is divided into a side facing the ball and a side facing the wall, due to the similar dimensionless bubble-ball distance, the deformation of the bubble facing the ball side is also very similar and is not affected by the wall; while on the side facing the wall, due to the different dimensionless bubble-wall distances, the two have a significant difference in morphology. In Fig. 12(A), the bubble's wall side does not come into contact with the wall throughout the entire expansion and collapse process, except for the final state, whereas in Fig. 12(B), it comes into contact with the wall during the expansion phase, but this does not affect the deformation of the bubble facing the ball side. This indicates that there is a proximity principle in bubble deformation, meaning that the deformation of the bubble wall is only related to the boundary conditions closest to it and is independent of the boundary conditions on the opposite wall side.

**Fig. 13:**
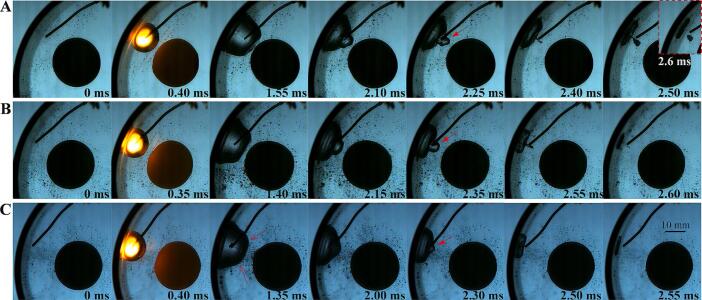
The process of generating toroidal jets outside the cavitation bubble. (A) $\gamma_{\mathrm{ball}}^{\text{bubble}}=0.64$, $\gamma_{\mathrm{tube}}^{\text{bubble}}=0.38$; (B) $\gamma_{\mathrm{ball}}^{\text{bubble}}=0.78$, $\gamma_{\mathrm{tube}}^{\text{bubble}}=0.30$; (C) $\gamma_{\mathrm{ball}}^{\text{bubble}}=1.14$, $\gamma_{\mathrm{tube}}^{\text{bubble}}=0.30$.

In the interaction of bubble-ball-wall, the toroidal jet is the most significant factor that changes the shape of bubbles. When the jet penetrates the bubble and enters its interior, it is not easy to observe. Therefore, studying weak annular jets first is a better choice. Fig. 13 shows three similar distributions with only minor variations. It can be seen from the figure that in the early stage of bubble expansion, the side of the bubble wall facing the elastic ball is slightly convex compared to the spherical shape, and in the later stage of bubble expansion, the side of the bubble wall facing the elastic ball is concave compared to the spherical shape, forming a toroidal protrusion on outer side of the ball-bubble-wall centerline. This protrusion has a faster bubble wall speed than other positions during the collapse process, thus forming a concave trend (toroidal jet). Because the bubble wall speed on the ball-bubble-wall centerline is slower than the surrounding bubble wall speed, it evolves into a protrusion. As the dimensionless bubble-ball distance decreases, the protrusion and jet become more pronounced. In Fig. 13(B), the residual of the collapsed protrusion can be seen, indicating that the protrusion has become a relatively independent part. In Fig. 13(A), it is shown that the jet divides the bubble into two parts (the outer toroidal bubble and the inner conical bubble). Comparing with the interaction between a bubble and a hard ball or a bubble and an elastic ball in an unrestricted space, it is found that the toroidal jet and protrusion are related to the curvature of the ball, and the wall effect intensifies the toroidal jet, especially in the later stages of bubble collapse, as the side of the bubble wall facing the ball gets closer to the tube wall.

**Fig. 14:**
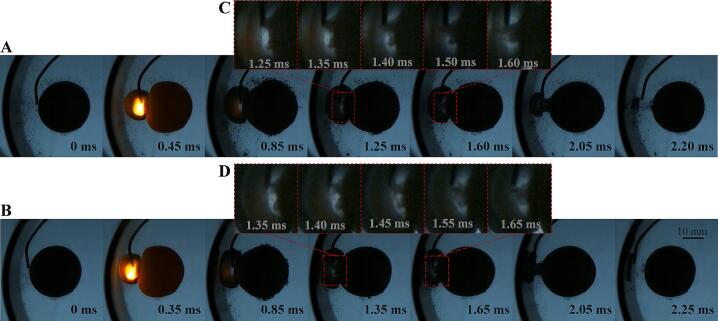
The process of generating toroidal jets inside the cavitation bubble (jet convergence). (A) $\gamma_{\mathrm{ball}}^{\text{bubble}}=0.00$, $\gamma_{\mathrm{tube}}^{\text{bubble}}=1.20$; (B) $\gamma_{\mathrm{ball}}^{\text{bubble}}=0.00$, $\gamma_{\mathrm{tube}}^{\text{bubble}}=0.78$; (C) and (D) Detail view of the toroidal jets.

**Fig. 15:**
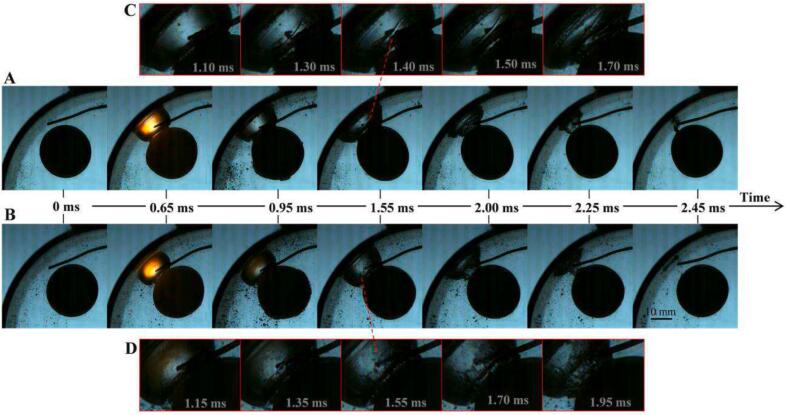
The process of generating toroidal jets inside the cavitation bubble (jet divergence). (A) $\gamma_{\mathrm{ball}}^{\text{bubble}}=0.26$, $\gamma_{\mathrm{tube}}^{\text{bubble}}=0.44$; (B) $\gamma_{\mathrm{ball}}^{\text{bubble}}=0.26$, $\gamma_{\mathrm{tube}}^{\text{bubble}}=0.64$; (C) and (D) Detail view of the toroidal jets.

Fig. 14 and Fig. 15 show the process of generating a circular jet that penetrates the bubble inside. Under the illumination of transmitted light, the leading edge of the jet inside the bubble can be seen. When the electrode is tightly attached to the ball wall, a convergent annular jet is generated (as shown in Fig. 14 (C) and (D)), and at a certain dimensionless bubble-sphere distance, a divergent annular jet can be generated (Fig. 15 (C) and (D)). Although the deformation of the bubble surface cannot be observed during the initial formation of the jet, it is speculated that its formation mechanism should be the same as the formation mechanism of the annular jet in Fig. 13, except for differences in the direction and intensity of the jet. The dimensionless bubble-ball distance is a key factor affecting the direction of the jet.

#### 3.2.3 Distribution-3

**Fig. 16:**
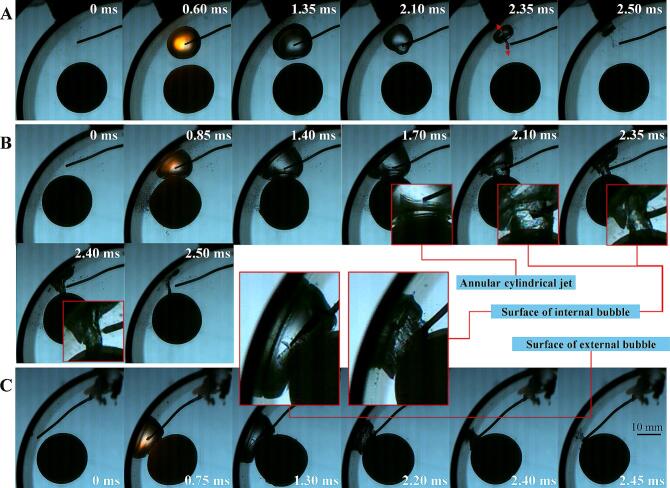
The generation and annihilation of the toroidal jet inside the bubble (β≠0). (A) $\gamma_{\mathrm{ball}}^{\text{bubble}}=0.97$, $\gamma_{\mathrm{tube}}^{\text{bubble}}=0.94$, *β* = 38°; (B) $\gamma_{\mathrm{ball}}^{\text{bubble}}=0.50$, $\gamma_{\mathrm{tube}}^{\text{bubble}}=0.52$, *β* = 42°; (C) $\gamma_{\mathrm{ball}}^{\text{bubble}}=0.36$, $\gamma_{\mathrm{tube}}^{\text{bubble}}=0.19$, *β* = 19°.

When the bubble is located in the sensitive zone of the elastic ball and also in the sensitive zone of the tube wall, and the ball-wall angle *β* ≠ 0, in other words, when the bubble is positioned at the corner of the elastic ball and the curved wall, and the bubble-ball interaction and bubble-wall interaction are not in the same direction, the contributions of the elastic ball and the curved wall to the bubble deformation can be distinguished by the direction of the bubble deformation. Fig. 16(A) shows the process of forming a bubble protrusion due to the weak toroidal jet when the dimensionless bubble-ball distance is relatively far. It can be observed that the protrusion faces the elastic ball, indicating that the protrusion is caused by the toroidal jet induced by the elastic ball. At *t =* 2.35 ms, the bubble collapse produced a microjet facing the wall (along the bubble-tube centerline), which suggests that this jet is not the result of the toroidal jet but rather the effect of the wall. As the dimensionless bubble-ball distance decreases, the toroidal jet becomes stronger and narrower, resulting in the formation of an annular sylindrical jet (as shown in Fig. 16(B)). It can be seen from the figure that at *t =* 0.85 ms, a protrusion was formed on the side of the bubble facing the ball, and the concave part between the protrusion and the main body of the bubble is the toroidal jet. When *t =* 1.4 ms, the toroidal jet had already penetrated the interior of the bubble, and by *t =* 1.70 ms, the toroidal jet (annular sylindrical jet) was fully developed, dividing the bubble into a cylindrical bubble inside the jet and a hollow cylindrical bubble outside the jet. As the outer hollow cylindrical bubble begins to collapse, the liquid jet merges with the surrounding fluid, and the surface of the inner cylindrical bubble is revealed (*t =* 2.10, 2.35, 2.40 ms). It can be observed from the figure that the direction of the toroidal jet and the direction of the cylindrical bubble inside the jet are consistent with the bubble-ball centerline, indicating the dominant role of the elastic ball in the generation of the toroidal jet. In the absence of the curved wall, a penetrating toroidal jet cannot be produced, indicating that in addition to the strengthening effect of bubble collapse on the toroidal jet, the wall's enhancement of disturbance on the bubble surface is also an important factor in the formation of the annular sylindrical jet. Fig. 16(C) shows an even more extreme case where both the dimensionless bubble-wall distance and the dimensionless bubble-ball distance are smaller, and it can be observed that a strong jet-like toroidal jet is generated inside the bubble. After the outer layer of the bubble collapses, the surface of the inner bubble is exposed, revealing a mottled striped surface, indicating that the thickness of the toroidal jet is not uniform. The outer layer of the bubble collapses first, followed by the inner layer, which is consistent with previous research findings.

**Fig. 17:**
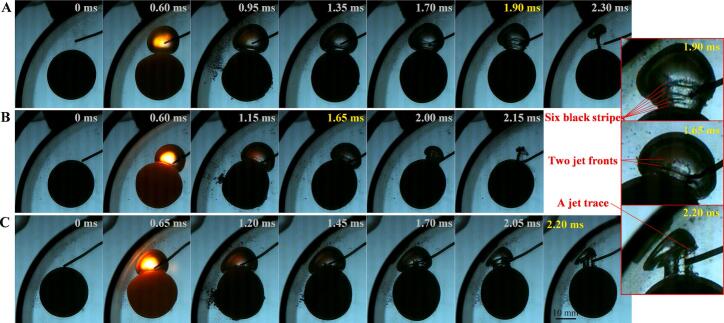
The multi-layered toroidal jet inside the bubble. (A) $\gamma_{\mathrm{ball}}^{\text{bubble}}=0.46$, $\gamma_{\mathrm{tube}}^{\text{bubble}}=1.22$, *β* = 43°; (B) $\gamma_{\mathrm{ball}}^{\text{bubble}}=0.00$, $\gamma_{\mathrm{tube}}^{\text{bubble}}=2.12$, *β* = 62°; (C) $\gamma_{\mathrm{ball}}^{\text{bubble}}=0.00$, $\gamma_{\mathrm{tube}}^{\text{bubble}}=1.20$, *β* = 48°.

A significant feature of the interaction between bubble, ball and curved wall is that in some cases, multi-layered toroidal jets can be generated inside the bubbles. The interaction between a cavitation bubble and an elastic plane can produce a toroidal jet, the interaction between a cavitation bubble and a ball can produce a toroidal jet, and the interaction between a cavitation bubble and an elastic ball, enhanced by the wall effect, can produce multi-layered toroidal jets, as shown in Fig. 17. In Fig. 17(A), at *t =* 1.7 ms, two leading edges of toroidal jets can be observed. At *t =* 1.90 ms, there are six dark lines can be seen, with three should be the leading edges of the jets and the other three should be the roots of the jets. Therefore, in fact, three nested toroidal jets have already formed before the collapse of the bubble. Fig. 17(B) shows two nested toroidal jets with significantly different diameters, where the inner jet has a smaller diameter and a serrated leading edge, clearly indicating a splash-type toroidal jet. There is a focused annular jet inside Fig. 17 (C), and a very shallow toroidal jet outside (the jet front and root are coincident). As mentioned earlier, due to the tendency of bubbles to collapse towards the center line of the ball-tube, the bubble wall on the right side of the bubble has a faster velocity, so that the right bubble wall passes over the jet before the internal jet dissipates (*t =* 1.70, 2.05, 2.20). The interaction between the liquid jet and the gas–liquid interface produces a large number of microbubbles in the original liquid jet area, forming a jet trace. This is another strong evidence for the existence of liquid toroidal jets.

### 3.3 Multi-layered nested toroidal jets

**Fig. 18:**
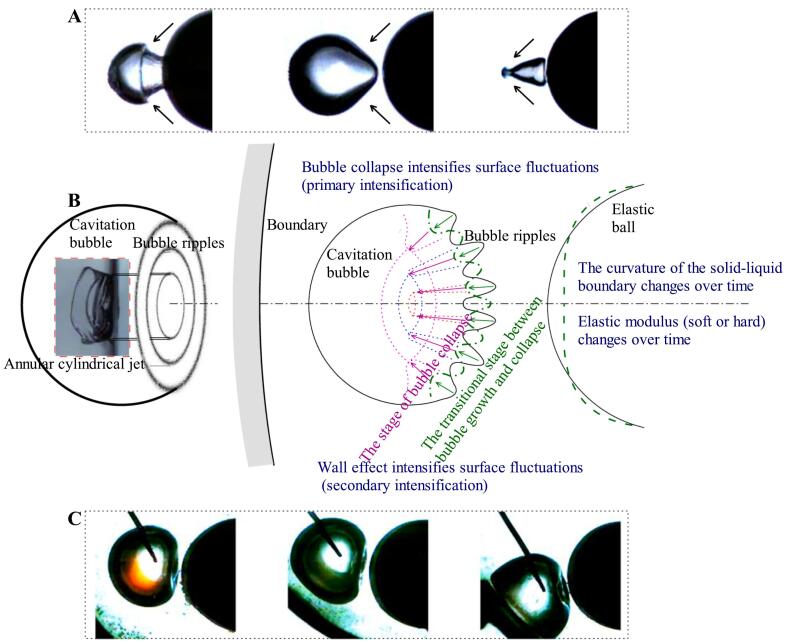
Bubble ripples dynamics of multi-layered toroidal jets formation in bubble-ball-wall interaction. (A) Special cases of bubble ripples (hard ball); (B) Schematic diagram; (C) Snapshots of bubble ripples (elastic ball).

During the interaction of cavitation bubble-elastic ball-curved wall, bubble ripples can be produced on the side of the bubble facing the elastic ball (as shown in Fig. 18(C)). Multi-layered nested toroidal jets are evolved from these bubble ripples. The formation and development of bubble ripples can be divided into three stages (as shown in Fig. 18(B)). In the early stage of bubble expansion, bubble ripples are very simple. When the bubble expands, the resistance of the bubble wall on the ball side is smaller (the ball behaves as soft), and the bubble wall on the ball side is slightly convex relative to the normal spherical shape. The transition stage between bubble expansion and collapse, also known as the maximum volume stage: Bubble ripples become more complex, forming multiple layers of concentric ripples. There are four main factors affecting these concentric ripples: 1. In the early stage of bubble expansion, the convex part on the line connecting the center of the bubble and the center of the ball rebounds in the opposite direction and spreads outwards; 2. The elastic ball begins to behave as soft near the line connecting the center of the bubble and the center of the ball, until it exceeds the elastic deformation threshold and then behaves as hard. It then behaves as soft when the bubble contracts, and finally behaves as hard when it reaches its limit. This wave of soft-hard transitions (elastic modulus wave) propagates from the bubble-ball centerline on the surface of the ball towards the periphery; 3. The deformation of the ball changes the curvature of the liquid–solid interface, and this curvature wave also propagates from the centerline of the bubble-ball towards the surroundings. The surface tension waves of the bubble, the elastic modulus waves of the ball, and the curvature waves of the ball work together, producing multi-layered bubble ripples during the transition stage from the end of bubble expansion to the beginning of bubble collapse, that is, during the maximum volume stage of the bubble. At this time, the bubble ripples show that the originally concave parts tend to become convex, and the originally convex parts tend to become concave. As the bubble enters the collapse stage, it rapidly collapses, and its volume decreases rapidly (primary intensification), coupled with the wall effect (secondary intensification), the bubble ripples are quickly intensified. The originally concave parts accelerate inward, while the originally convex parts cremains relatively unchanged, thus the concave parts evolve into multi-layered nested toroidal jets. Some common bubble shapes can be considered as special cases of bubble ripples (as shown in Fig. 18(A)).

## 4 Conclusion

The interaction between a cavitation bubble and an elastic ball in a hard boundary tube was investigated experimentally using single-electrode periodic discharge bubble generation technology and high-speed photography. The paper focused on the translational motion of the elastic ball and the formation mechanism of the multi-layered nested toroidal jets in cavitation bubble.

(1) As the dimensionless bubble-ball distance increases, the cavitation bubble and the hard ball exhibit a ‘repulsion-attraction–repulsion-attraction’ process, while the cavitation bubble and the elastic ball show a ‘repulsion-attraction’ process. This is mainly due to the combined effects of the expansion ejection effect, the reverse thrust of liquid jet and the secondary Bjerknes force of cavitation bubble and its rebound bubble.
(2) The radial vibration of the elastic ball causes a secondary Bjerknes force attraction effect between the ball and the wall, similar to that between an acoustic bubble and a wall.
(3) In the interaction of “cavitation bubble-elastic ball-curved wall,” there is a state of equilibrium stability where the centerline of the “bubble-ball” coincides with the centerline of the “bubble-wall”. This is the result of the three forces with different starting and ending points—the “bubble-wall” secondary Bjerknes force, the “ball-wall” secondary Bjerknes force, and the “bubble-ball” interaction force—reaching a condition of equilibrium.
(4) The surface tension waves of the bubble, the elastic modulus waves and the curvature waves of the elastic ball work together to form cavitation bubble ripples. Under the primary intensification of the bubble's rapid collapse and the secondary intensification of the wall effect, the bubble ripples are reinforced, leading to the formation of multi-layered nested toroidal jets.

## CRediT authorship contribution statement

**Yanyang Liu:** Writing – original draft, Visualization, Software, Investigation, Data curation. **Jing Luo:** Visualization, Investigation, Data curation. **Lixin Bai:** Writing – original draft, Supervision, Methodology, Investigation, Funding acquisition, Conceptualization. **Jiankun Hu:** Supervision, Resources, Methodology, Conceptualization.

## Declaration of competing interest

The authors declare that they have no known competing financial interests or personal relationships that could have appeared to influence the work reported in this paper.
